# Impact of *Kelussia odoratissima* Mozaffarian lipid profile and fasting blood sugar in hyperlipidemia patients

**Published:** 2015-07-22

**Authors:** Marzieh Kafeshani, Mahmoud Mirhosseini, Ali Momeni, Rahmatollah Rabiei, Mahmoud Rafieian-Kopaei

**Affiliations:** ^1^Nutrition and Food Science School, Isfahan University of Medical of Sciences, Isfahan, Iran; ^2^Medical Plants Research Center, Shahrekord University of Medical Sciences, Shahrekord, Iran

**Keywords:** Hyperlipidemia, Human, Kelussia odoratissima, Triglyceride, Cholesterol

## Abstract

**Introduction:** Hyperlipidemia is associated with increased risk of cardiovascular disease. Each type of medication works differently and has different types of side effects. Flavonoids are a group of polyphenolic compounds with antioxidant properties that help reducing the cardiovascular risk factors. *Kelussia odoratissima* is a flavonoid containing plant.

**Objectives:** The aim of this study was investigating the effect of this herb on lipid and glucose profile in hyperlipidemia patients.Patients and Methods: This study performed on 61 hyperlipidemia patients. They assigned in control and intervention groups. The control group received 40 mg/day of lovastatin and intervention group received 40 mg/day of Lovastatin plus 2 g/day powder of *Kelussia odoratissima*. Before, two weeks and 1 month after the beginning of the study, cholesterol, triglyceride (TG), low density lipoprotein cholesterol (LDL-C), very low density lipoprotein cholesterol (VLDL-C), high density lipoprotein cholesterol (HDL-C), and fasting blood sugar (FBS) were measured. Data were analyzed by variance analysis with repeated measures, chi-square and t tests.

**Results:** The reduction rate of cholesterol, TG, VLDL-C and LDL-C was similar in the control and intervention groups. The HDL-C rate was higher in intervention group compared to control group (*P* < 0.05). The mean LDL/HDL ratio decreased during the study (*P* < 0.001); however, there was not any significant difference between the two groups (*P* > 0.05). The mean of FBS did not change between and within groups (*P* > 0.05).

**Conclusion:** *Kelussia odoratissima* did not have a desirable effect on serum lipid profile and FBS in hyperlipidemic patients that use lovastatin, but is able to increase HDL-C significantly

**Implication for health policy/practice/research/medical education:**

In a study on 61 hyperlipidemia patients who were treated with 40 mg/day of lovastatin plus 2 g/day powder of Kelussia odoratissima, this herb did not have a desirable effect on serum lipid profile and fasting blood sugar (FBS) in hyperlipidemic patients, however, this herb had the capability to increase high density lipoprotein cholesterol (HDL-C) significantly.

## Introduction

Dyslipidemia is a high level of lipids (cholesterol, triglycerides [TGs], or both) carried by lipoproteins in the blood. This term includes hyperlipoproteinemia (hyperlipidemia), which refers to abnormally high levels of total cholesterol, low density lipoprotein (LDL-C) or TG, as well as an abnormally low level of high density lipoprotein (HDL-C). Hyperlipidemia can have a serious effect on health (1). It can significantly increase the risk of developing cardiovascular disease, including disease of blood vessels supplying the heart (coronary artery disease), brain (cerebrovascular disease), and limbs (peripheral vascular disease) (2). Diet, physical activity and drugs are main treatments for hyperlipidemia (3). The available drugs have some side effects, so recently attention has been turned to the use of medicinal plants that have usually less side effects and are accepted by people as an alternative therapy since the earliest times (4). Traditional herbal medicines are getting significant attention in global health debates (5,6). One of the herbs is *Kelussia odoratissima*. This plant is from Umbelliferae family and is harvested in April and in a restricted region in west of Iran. *Kelussia* is a perennial and very aromatic. This plant is used to treat some diseases such as rheumatologic disease, hypertension, blood lipid abnormalities, common cold, and stomachache in local area. Studies have shown that it has anti-diabetes, anti-inflammation, and anti-allergy effects due to its flavonoids (7). Butyl phthalide and ligustilide are the main components of *Kelussia odoratissima*. Phthalides constitutes 70% of the plant extract with the effect of prostaglandin F2α inhibition, liver disorders therapy and blood viscosity reduction and the ligustilide leads to relax vessels of rat (8). Also the effectiveness of this plant in treating hyperlipidemia is verified in animal studies but no research has been done relating to this effect in human. So, the aim of this study was evaluating this effect in clinical practice.

## Patients and Methods

This clinical trial study carried out on 61 hyperlipidemic patients. The patients had no liver, kidney or infectious diseases and referred to the Internal Clinic of Shahrekord University of Medical Sciences. Every patient with at least one of the following criteria in the serum lipid profile was recognized as a hyperlipidemic patient (9). A cholesterol level above 240 ml/dl, TG level above 200 ml/dl, HDL-C level below 50 ml/dl and LDL-C level above 130 ml/dl.

They assigned into two intervention and control groups ordered by their arrival. Among all the patients, 30 were placed in the intervention group, and 31 in the control group. Patients were asked to avoid making any change in their diet during the study and they were asked to inform the researchers in case of any change in their diet or their medications, to be excluded. Patients of the intervention group received lovastatin (40 mg/day) along with packages including 60 g of *Kelussia odoratissima* plus a container having the capacity of 2 g. The patients of this group were asked to take 2 g of the powder in 150 cc low-fat yogurt at the lunch time without making any change in their food diet. The control group was asked to have lovastatin (40 mg/day) along with 150 cc low-fat yogurt at the lunch time. Two weeks and also 1 month after starting the study, all the patients underwent biochemical tests including cholesterol, TG, LDL-C, HDL-C, VLDL and fasting blood sugar (FBS) at the same laboratory (by BT3000 auto-analyzer) and the results were registered in separate files related to each patient. The personnel of the laboratory did not have any information about the studied groups.

### Ethical issues

1) The research followed the tenets of the Declaration of Helsinki; 2) Informed consent was obtained; and 3) the proposal of the research was approved in the Ethics Committee of Shahrekord University of Medical Sciences.

### Statistical analysis

After completing the above stages, the obtained data were analyzed by variance analysis with repeated measures, chi-square and *t* tests. *P* value with rate of less than 0.05 was considered as significant level.

## Results

In this study, 30 patients participated as the intervention group and 31 as the control group. Chi-square test did not show any significant difference between the two groups regarding gender, being urban or villager, education level, the type of consumed oil in diet, the type of daily activities and the drug history (*P*>0.05).

The results showed that the reduction rate of cholesterol, TG, VLDL-C and LD-CL was similar in the control (lovastatin, 40 mg/day) and intervention (lovastatin 40 mg/day and *Kelussia odoratissima* powder, 2 g/day) groups. The difference HDL-C rate was significant before and after study in the two groups; this difference increased in the intervention group with the average rate of 5.5 units and decreased in control group with the average rate of 2.5 units (*P*<0.05).

The mean LDL/HDL ratio decreased during the study (*P*<0.001); however, there was not any significant difference between the two groups (*P*>0.05). The mean of FBS did not change and the two groups had no significant difference (*P*>0.05; Table 1).

**Table 1:** The changes of biochemical factors^a^ in hyperlipidemic patients after taking *Kelussia odoratissima* in the patients who take lovastatin

**Variable**	**Groups**					
	**Control**			**Case**		
	**Before intervention**	**2 weeks later**	**1 month later**	**Before intervention**	**2 weeks later**	**1 month later**
Cholesterol	246.67±46.28	176.16±43.61	171.70±33.10	247.10±47.99	177.30±57.02	180.23±45.29
TG	265.29±174.87	177.74±85.08	191.03±85.66	305.36±218.98	235.40±275.52	228.56±206.86
LDL-C	138.86±42.08	89.49±41.75	83.51±35.63	138.28±44.20	74.44±51.53	88.60±40.71
VLDL	58.52±36.60	34.16±13.29	37.90±17.27	62.36±41.68	40.60±26.45	40.66±24.23
HDL/LDL	2.60±0.61	1.70±0.71	1.70±0.79	2.97±1.1	1.74±1.02	1.75±0.90
FBS	116.79±45.62	121.16±50.81	111.93±42.67	116.26±42.17	115.46±53.28	120.96±58.52

Abbreviations: LDL-C: low density lipoprotein cholesterol; HDL-C: high density lipoprotein cholesterol; VLDL-C: very low density lipoprotein cholesterol; FBS, fasting blood sugar. *P*<0.001 during the study in both groups for all variables except FBS. *P*>0.05 between the two groups during the study in all variables, case group receiving Lovastatin (40 mg/day) and *Kelussia odoratissima* Mozaffarian (2 g/day), control group receiving Lovastatin (40 mg/day). ^a^Due to the existence of difference between the two groups in the rate of HDL before consuming the drug, the difference in the rate of HDL was analyzed at the beginning and the end of the study, so that in the case group it has increased 5.5 units and in the control group it has decreased 2.5 units (*P*<0.05).

## Discussion

The results of study showed that cholesterol, LDL-C, VLDL-C and TG had a significant reduction during the study. However, this rate of reduction was not statistically significant in control and intervention group. The difference HDL-C rate was significant before and after study in the two groups; this difference increased in the intervention group with the average rate of 5.5 units and decreased in control group with the average rate of 2.5 units. The mean of LDL/HDL ratio decreased during the study. However, there was no significant difference between the two groups. The mean of FBS had no change during the study and both groups were similar in this respect.

Different studies have shown that *Kelussia odoratissima* have flavonoid combinations (10). The fraction analysis of the total extract of the plant has shown the routine existence of 3, 4, 7-trihydroxyflavone, caffeic acid, and phthalide. Since mentioned flavonoids are all in form of aglycone, they have fast intestinal absorption due to the specific structure (7). Therefore, the lipid decrease was expected after the use of *Kelussia odoratissima* Mozaffarian. Also caffeic acid in this plant has antioxidant properties which can decrease harmful lipids through inhibition of cholesterol biosynthesis. The cholesterol synthesis regulation is normally done by HMG-CoA. The conversion reaction of HMG-CoA to mevalonate takes place by the mediation of HMG-CoA reductase and NADPH (Nicotine amide adenine dinucleotide phosphate) that is the main affecting point of statins antihypercholesterolemic agents (11). One explanation for our result is taking lovastatin as treatment in both intervention and control groups. Since researchers could not deprive patients from taking drug in the human study due to ethical issues. Taking lovastatin will lead the level of lipid to a normal level. This issue causes that the consumption of the plant cannot decrease the lipid easily.

Flavonoids increase the level of HDL-C through increasing ApoA-I synthesis; therefore, increasing the level of HDL-C is probably due to existence of flavonoid contents of *Kelussia odoratissima* in the intervention group (12).

The LDL/HDL ratio decreased. This ratio is highly significant in predicting the rate of coronary diseases. This relation can be justified with the roles suggested for LDL-C in transferring cholesterol to tissues and the role of HDL-C in the reverse transfer of cholesterol (13).

Herbal antioxidants may also have insulin like effect and increase the absorption of glucose in peripheral tissues (14). Moreover, antioxidants increase the secretion of insulin and decrease the amount of glucose in the body through affecting beta cells. However, in this study, taking *Kelussia odoratissima* did not make any change in patients’ blood glucose in spite of expectations. This issue can be probably due to the limitations that exist despite the necessary trainings of patients on controlling their diet and activity. This issue needs further research anyway. *Kelussia odoratissima* increased the HDL-C level which is an indicator for increase in serum antioxidant capacity. Oxidative stress has a predominant contribute to cardiovascular diseases, especially atherosclerosis as well as most of hard curable diseases cancer, diabetes and infections (13). Antioxidants have been shown to counteract the toxic effects of free radicals preventing a lot of free radicals induced diseases. Therefore, increase in antioxidant capacity of the body by this plant may promote beneficial effects in these complications. There are a lot of medicinal plants which have antioxidant activity which may have the same effects on these diseases (15).

Therefore, performing long-term studies on the effect of normal antioxidants and especially *Kelussia odoratissima* on lipid and glucose of hyperlipidemic patients seems necessary.

## Conclusion

In this study adding the *Kelussia odoratissima* powder to the diet of hyperlipidemic patients did not cause any considerable difference except increasing HDL-C in comparison with patients who took their routine medications. Therefore, performing long-term studies on the effect of normal antioxidants and especially *Kelussia odoratissima* on lipid and glucose of hyperlipidemic patients seems necessary.

## Limitations of the study

This study was conducted on a small group of patients. We suggest larger studies on this subject.

## Authors’ contribution

All authors wrote the manuscript equally.

## Conflicts of interest

The authors declared no competing interests.

## Ethical considerations

Ethical issues (including plagiarism, misconduct, data fabrication, falsification, double publication or submission, redundancy) have been completely observed by the authors.

## Funding/Support

This study was supported by a grant from Shahrekord University of Medical Sciences.
